# Nasal biomarker testing to rule out viral respiratory infection and triage samples: a test performance study

**DOI:** 10.1016/j.ebiom.2025.105820

**Published:** 2025-06-20

**Authors:** Julien A.R. Amat, Sarah N. Dudgeon, Nagarjuna R. Cheemarla, Timothy A. Watkins, Alex B. Green, H. Patrick Young, David R. Peaper, Marie L. Landry, Wade L. Schulz, Ellen F. Foxman

**Affiliations:** a Department of Laboratory Medicine, Yale School of Medicine, New Haven, CT, USA, 06520; b Department of Immunobiology, Yale School of Medicine, New Haven, CT, USA, 06520; c Department of Paediatrics, Yale New Haven Children's Hospital, New Haven, CT, USA, 06520; d Department of Internal Medicine, Yale School of Medicine, New Haven, CT, USA, 06520

**Keywords:** Respiratory virus screening, COVID-19, CXCL10, IP-10, Host biomarker, Host response diagnostics

## Abstract

**Background:**

The COVID-19 pandemic revealed an urgent need for practical screening tests to rule out respiratory virus infection, both for managing outbreaks and for routine screening in high-risk settings. PCR is the gold standard test for respiratory virus diagnosis but requires specialised equipment, uses different assays for each virus, and often excludes emerging viruses. The goal of this study was to evaluate a pan-viral host biomarker to rule out respiratory virus infection. We used CXCL10, a cytokine induced in the nasal mucosa in response to diverse respiratory viruses.

**Methods:**

We compared immunoassay for CXCL10 to respiratory virus PCR panel results in 1088 nasopharyngeal samples from adults and children with an overall viral prevalence of 32.6% by PCR. Using this data, we mathematically modelled the impact of CXCL10 biomarker testing on patient triage and resource savings at different viral prevalences. We also explored clinical features associated with false negatives using automated data extraction from electronic medical records.

**Findings:**

CXCL10 accurately predicted virus positivity (A.U.C. 0.87, 95% C.I. 0.85–0.90). Mathematical modelling predicted that CXCL10 screening would enable a significant reduction in PCR testing, especially when viral prevalence is low (e.g. 92% of samples testing negative when viral prevalence is 5%, NPV = 0.975). Outlier analysis identified specific chemotherapeutic drugs and low viral load as features associated with false negatives.

**Interpretation:**

These results demonstrate the utility of a nasopharyngeal biomarker to rule out respiratory infection, with potential applications in outbreak management and/or routine screening in high-risk settings.

**Funding:**

Yale-New Haven Hospital Innovation Fund and 10.13039/100000002NIH.

**Research in context**

**Evidence before this study**

To identify other studies evaluating biomarker-based approaches for respiratory virus screening, we searched PubMed from database inception up to February 18, 2025, using the following search terms “respiratory AND virus AND biomarker AND diagnosis AND (patient OR triage)”, filtering to exclude non-human research, preprints and reviews. Of the 1356 publications retrieved, most emphasised the use of biomarkers to distinguish between mild and severe infections, while others focused on discriminating viral from bacterial infections (72% and 14%, respectively). A subset of reports (n = 184) used clinical data from hospitalised patients to predict infection status. Only 119 reports focused on biomarkers of virus status for triage, including detection of volatile organic compounds in exhaled breath, combinations of host proteins in blood, or bronchoalveolar lavage. To our knowledge, no previous studies have investigated the effectiveness of a single nasopharyngeal biomarker.

**Added value of this study**

This study presents proof-of-concept data that nasopharyngeal CXCL10 testing could be used as a practical and cost-effective strategy to identify virus-negative individuals with high negative predictive value, especially when viral prevalence is low. We also use automated data extraction from the electronic medical record to explore clinical features associated with false negative biomarker tests to identify possible exclusion criteria.

**Implications of all the available evidence**

Nasopharyngeal CXCL10 testing has potential as a practical approach to rule out viral respiratory infection when viral prevalence is low, which could be used for triage and conservation of resources during an epidemic and/or for routine respiratory virus screening in high-risk settings (e.g. hospitals, institutional care facilities, military barracks).

## Introduction

The COVID-19 pandemic motivated advances in diagnostic testing for respiratory viruses but also revealed unmet needs, including the necessity for better screening strategies to rule out viral respiratory infection. First, the pandemic highlighted that human populations regularly interface with a highly dynamic pathogen landscape, compelling urgent preparation for future respiratory virus epidemics.^1^^,^^2^ Early mitigation strategies for an emerging respiratory virus include isolation and quarantine of infected individuals to limit viral spread, especially when medications and vaccines have not yet been developed and pre-existing immunity is limited or absent.^3^ Rapid and accurate screening methods are critical, not only to reduce transmission, but also to identify and triage infected patients amidst limited healthcare resources, and to reduce the socioeconomic disruptions imposed by large-scale or prolonged quarantines of uninfected individuals.^4^ Second, the pandemic motivated increased viral surveillance in hospital and institutional care settings and revealed the importance of asymptomatic transmission events in spreading viruses to vulnerable patients.^5^ Evidence that SARS-CoV-2 and other respiratory viruses can cause infections and transmission even when asymptomatic further highlights the limitations of symptom-based screening.^6^, ^7^, ^8^ These observations compel development of efficient and accurate screening tests for hospitals, institutional care facilities, and other settings for which preventable viral transmission has serious consequences (e.g. healthcare facilities, military barracks, etc.).

Polymerase chain reaction (PCR) and other nucleic acid amplification tests (NAAT) are sensitive and specific for respiratory viruses, and ultimately became the standard of care for COVID-19 screening, but the pandemic revealed important limitations. First, PCR tests are virus-specific and new tests must be developed for emerging viruses. PCR testing for COVID-19 was not widely available in the US until March 2020 and continued to be challenging due to long turn-around-times, high costs, need for specialised equipment, and supply chain limitations during the first year of the pandemic.^9^^,^^10^ Once widely available, routine screening by PCR/NAAT was deployed for infection control in hospitals, assisted care facilities, and schools, resulting in effective but costly large-scale screening. Another limitation of PCR/NAAT for infection control is the need for a different test for every virus, making it costly and impractical as a screening test to rule out viral respiratory infection when multiple viruses are circulating.

Host biomarkers have recently gained interest as a complementary approach in infectious disease diagnostics, and both blood and nasal biomarkers have been proposed for diagnosis of respiratory infections.^11^, ^12^, ^13^, ^14^, ^15^, ^16^, ^17^ We previously identified the interferon-inducible protein CXCL10 as a nasopharyngeal biomarker of diverse viral respiratory infections, suggesting possible utility as a screening test.^16^ In contrast to PCR, which detects genetic sequences specific to individual viruses, viral sensors of the innate immune system detect features common to all viruses, such as structures present in viral, but not human, genomes.^18^ Such biomarker-based assays, while less pathogen-specific than PCR, offer advantages over PCR tests in being able to capture many viruses with a single test, including unexpected or emerging viruses. To this end, we previously showed that nasopharyngeal CXCL10 could identify patients with unexpected respiratory viruses, including undiagnosed cases of SARS-CoV-2 from early in the COVID-19 pandemic prior to the availability of PCR tests.^19^ These results indicate the potential for this biomarker to be used as a screening test even for a novel emerging virus. Given the high cost of comprehensive viral PCR panels, biomarker-based testing also offers a potentially cost-effective method to rule out viral respiratory infection when multiple viruses are circulating (e.g. winter respiratory virus season).

Here we evaluated the performance of nasopharyngeal CXCL10 screening to rule out viral respiratory infection in diverse patients and settings, benchmarking against comprehensive PCR testing for a panel of 15 commonly tested respiratory viruses. Our results show the utility of host response-based screening for public health management, the triage of patients, and the effective allocation of scarce testing resources when large-scale screening is indicated for infection prevention and control.

## Methods

### Clinical samples and metadata

Residual nasopharyngeal (NP) samples (n = 1088) and clinical metadata were obtained from the Yale–New Haven Hospital (YNHH) Clinical Virology Laboratory. Residual samples from adults (ages ≥18), had undergone virological PCR testing for 15 respiratory virus panel (RVP) as part of clinical care from January 1–31, 2020. Sixteen influenza-positive samples from adults tested on March 3–11, 2020 were also included to increase representation of influenza virus. Paediatric samples (ages <18) were from SARS-CoV-2 screening during June 3– July 2, 2021 and January 11–23, 2022 on patients with and without respiratory symptoms, and from 149 symptomatic paediatric patients tested from August 9–18, 2021 as previously described.^20^ Test results and clinical metadata were extracted from the laboratory information system and from the Yale–New Haven Health Computational Health Platform, which uses the Observational Medical Outcomes Partnership (OMOP) data model with data transformed from the Epic electronic health record (EHR).^21^ The data extract included demographic information, medical conditions, and drug exposures for one year prior to specimen collection. International Classification of Disease 10 (ICD-10) codes were used to define conditions and drugs which were grouped into therapeutic classes based on the anatomical therapeutic chemical (ATC) class. Race and ethnicity designations reported here are based on self-identification of patients during clinical care as recorded in the electronic medical record. All samples and data were de-identified prior to analysis. Use of residual samples and data was approved by the Yale Human Investigations Committee and determined not to require specific patient consent (Protocol #2000027656).

### Specimen handling

All samples were healthcare provider-collected nasopharyngeal (NP) swab specimens, collected with flocked swabs placed into 3 mL universal transport media (Viral Specimen Collection Kit, Copan Diagnostics, 3C064N). NP swabs were transported to the YNHH clinical laboratory. At the time of accessioning, swab-associated UTM underwent comprehensive respiratory virus testing or testing for SARS-CoV-2 based on physician orders for clinical care as described below, and residual swab-associated UTM was immediately frozen and stored at −80 °C (adult samples) or within three days of testing after storage at 4 °C (paediatric samples). For additional testing, UTM samples were thawed on ice and aliquoted. One aliquot was used for CXCL10 protein measurements as described below. For samples which did not undergo RVP testing at the time of accessioning, a separate aliquot was used for RVP testing at the time of thawing.

### Clinical virology testing and CXCL10 protein measurements

For comprehensive respiratory virus PCR testing, nucleic acids were isolated from 0.2 mL of UTM using the NUCLISENS (Boom method) on the EasyMAG instrument (BioMérieux; RRID: SCR_022666). Swab-associated UTM was tested for 15 respiratory viruses using a previously-described custom laboratory-developed respiratory virus PCR panel (RVP)^19^ which includes 6 duplex reactions (Influenza A/B, RSV A/B, rhinovirus/human metapneumovirus, human coronaviruses CoV-229E/OC43, human coronaviruses CoV-NL63/HKU1, parainfluenza 4/b-actin control), 1 triplex reaction (parainfluenza virus 1, 2, and 3), and 1 singleplex reaction (adenovirus). For SARS-CoV-2, PCR testing was done during clinical care, and viral cycle threshold (Ct) values were normalised across PCR testing platforms as previously described.^19^ For paediatric samples, the 15-virus RT-qPCR RVP was performed specifically for this study or for a prior study.^19^ CXCL10 protein concentration in NP swab-associated viral transport media was measured using a microfluidics-based ELISA assay on the automated ELLA™ instrument (BioTechne, San Jose, California) using the SimplePlex Human CXCL10/IP-10 Cartridge (BioTechne, Catalogue #: SPCKB-PS-000237), which determines the cytokine concentration based on three replicate measurements of the sample across 4 orders of magnitude as described previously.^19^^,^^22^

### Receiver operating characteristic curve (ROC) analysis

For ROC curve analysis, virus positivity by PCR was defined as a Ct value of 35 or 40 (the limit of detection) depending on the analysis, as indicated. When a viral coinfection was present, the lower of the two Ct values, indicating the virus with higher viral load, was used. To investigate ROC curves for detecting individual viruses, we restricted the analysis to individual viruses with >50 positives in the dataset (n = 100, 95, and 55 for RSV, rhinovirus, and SARS-CoV-2, respectively). For individual viruses, we created balanced datasets by using virus positives and an equal number of randomly selected virus-negative samples and calculated the mean ROC curve ±95% C.I. based on one thousand iterations (1000 bootstraps), using pROC v1.18.2 R software package.^23^^,^^24^

### Outlier analysis

For outlier analysis, we used virus-positive samples based on PCR (Ct ≤ 35) and assigned the biomarker result as “true positive” or “false negative” based on comparing the PCR and the biomarker test using a CXCL10 cutoff corresponding to a negative predictive value (NPV) of 0.90 (102 pg/mL). Using Pearson's Chi-square test for categorical features and a two-sample t-test for continuous variables, we compared the distribution of clinical features within the cohort to identify conditions and drug classes associated with false negative status. Outlier traits (p < 0.05) were evaluated for possible clinical relevance by a physician on the research team. Due to the large number of comparisons performed in this exploratory analysis, we did not correct for multiple comparisons but instead corroborated major results through other methods. To compare virus-positive samples associated with specific antineoplastics to virus-positive samples not associated with antineoplastic drug use, we compared the regression lines for the log_10_-transformed value of CXCL10 vs. Ct value by RT-qPCR for each group, using the analysis of covariance (ANCOVA) to compare the slopes and y-intercepts in GraphPad Prism software (RRID:SCR_002798). To explore the association of false negative tests with low viral load based on Ct value by RT-qPCR, Ct values for “true positives” and “false negatives” were compared using a student's t-test.

### Logistic regression modelling

We used generalised linear models (GLMs) with a binomial family distribution using the ‘glm’ function from the R software to assess the association between CXCL10 levels and viral infection.^24^ In this model, virus presence (binary outcome) was the response variable, and log_10_-transformed CXCL10 level was the predictor variable.

To evaluate test performance at viral prevalences relevant to a screening test (5–30% prevalence), simulated datasets were created as follows. Samples were first categorised as positive or negative based on PCR (Ct ≤ 35). Next, random subsampling (600 total observations) was performed using the ‘sample’ function from the R software package (RRID:SCR_001905) on each subset to create simulated populations at different viral prevalences (5%, 10%, 20%, or 30%).^24^

For each simulated dataset, a binomial logistic regression model was built as described above, allowing us to calculate test performance parameters and graph total negative tests, total positive tests, and false negative tests in populations with different virus prevalences across a range of test cutoffs (0.01–0.99 predicted probability of infection by 0.01 increment). The process was repeated over one thousand iterations (1000 bootstraps) to estimate test performance in populations at four viral prevalences (5%, 10%, 20%, or 30%).

NPV and the percentage of false negative tests were calculated as follows, with TN = true negatives, FN = false negatives:

$$NPV=\left(TN\right)/\left(TN+FN\;\right)$$

$$\%False\;negative\;tests=\left(FN\right)/\left(Total\;sample\;number\right)$$

### PCR positivity rates from COVID-19 testing 2020–2021

COVID-19 testing was performed on multiple platforms at YNHH during 2020-21 as previously described.^25^ Indication was captured in the EHR based on ask-at-order-entry questions and used to graph rates of viral positivity by test indication. Patients grouped under the term “contracts” represent scheduled, routine asymptomatic surveillance efforts largely at nursing homes and universities.

### Resource utilization and cost analysis

The estimated resource savings associated with CXCL10 pre-screening was based on the proportion of predicted negative patients (negative screening tests = PCR tests saved). Cost savings of CXCL10 pre-screening was estimated as follows:

$$Savings\;\left(\%\right)=100-\left(\left(\frac{\left(N_{}\times C_{CXCL10}\right)\;+\;\left(N_{pos}\times C_{PCR}\right)}{N_{}\times C_{PCR}}\right)\times 100\;\right)$$

In this equation, N = total number of samples, N_pos_ = Number of CXCL10 biomarker positive samples, C_CXCL10_ = cost of CXCL10 biomarker testing and C_PCR_ = cost of PCR testing in U.S. dollars. (N x C_CXCL10_) represents the total cost of CXCL10 testing to screen all samples, while (N_pos_ x C_PCR_) represents the cost of PCR testing of CXCL10-positive samples.

### Statistics

Graphing and statistical analyses used R Studio v4.2.2 software.^24^ Statistical tests including ROC analysis was performed using the pROC package (SCR_024286).^23^ To examine the relationship between log10-transformed CXCL10 concentration and PCR positivity, generalised linear model (GLM) was employed using the R glm () function.^24^ Statistical significance among the linear regressions was determined using ANCOVA analysis conducted in GraphPad Prism, while significance differences between two groups or more were calculated using student's t-test and Dunnett's test, respectively. Datasets were generated using the dplyr package (RRID:SCR_016708), while graphs were generated using the ggplot2 package (RRID:SCR_014601).^24^^,^^26^ This study used convenience residual samples from clinical testing, and sample size was determined as all available samples meeting inclusion criteria within specified date ranges. Detailed inclusion criteria are described in “Clinical samples and metadata”.

### Role of funding source

The funders of the study had no role in study design, data collection, data analysis, data interpretation, or writing of the report.

## Results

**Fig. 1:**
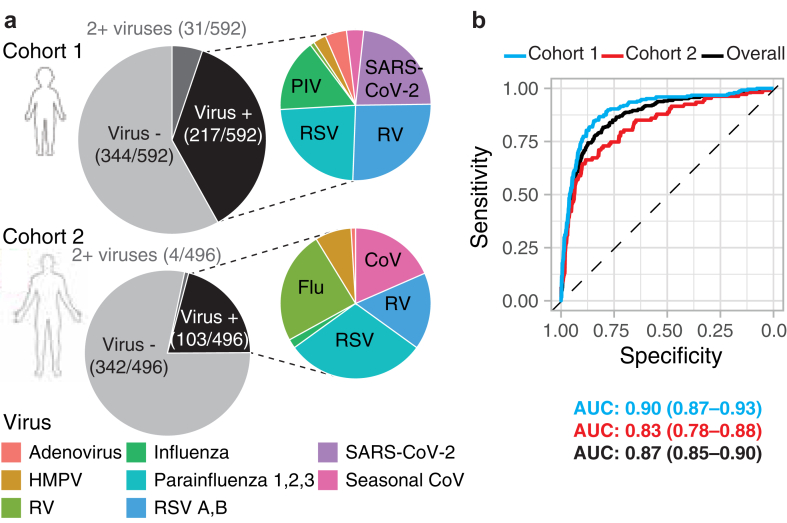
**Virus detection by PCR and nasopharyngeal CXCL10 in distinct patient cohorts**. **(a)** Viruses detected by PCR in nasopharyngeal swabs of 592 paediatric (cohort 1) and 496 adult (cohort 2) patients undergoing clinical virology testing from 2020 to 2022 for adenovirus, human metapneumovirus (HMPV), rhinovirus (RV), influenza viruses A and B (Flu), parainfluenza 1–3 (PIV), parainfluenza virus 4 (PIV4), RSV A and B (RSV), and seasonal coronaviruses CoV-229 E, OC43, NL63, and HKU1 (CoV), and SARS-CoV-2. **(b)** Receiver Operating Characteristic (ROC) curves depicting the prediction of virological status based on the log_10_-transformed CXCL10 protein concentration in cohorts 1 and 2 shown in blue and red respectively or combined (black). Area under the ROC curve (AUC) and 95% confidence intervals (CI) of each cohort or overall are shown.

To assess the use of nasopharyngeal CXCL10 as a screening test to rule out viral respiratory infection, we used 1088 residual nasopharyngeal (NP) swab samples from clinical testing at Yale–New Haven Hospital (YNHH), from children (cohort 1; n = 592, ages 0–18 years) and adults (cohort 2; n = 496, age >18 years) of both sexes (538 females; 550 males) and varied races (28 Asian; 226 Black or African American; 611 White; 223 other/unknown)and ethnicities (272 Hispanic; 801 Non-Hispanic, 6 other/unknown), which included residual samples which had been tested for respiratory viruses using a comprehensive respiratory virus panel (RVP) for clinical care or for another study.^20^ (Fig. 1, Table S1). Samples demonstrated high frequency viral positivity with 32.6% of samples testing virus-positive overall, including 41.9% of paediatric samples (248/592) and 21.6% of adult samples (107/496) (Fig. 1a, Table S1). The same seasonal viruses were observed in both cohorts, albeit at different proportions (Fig. 1a). SARS-CoV-2 was only present in the paediatric cohort since adult samples were collected prior to March 2020. Coinfections were detected in both groups, with higher rates in children (31/248; 12.5% vs. 4/107; 3.7% in cohorts 1 vs. 2; Fig. 1a).

Next, we examined test performance by age, race, sex, ethnicity, and virus. For virus-positive samples, we observed a strong positive correlation between the nasopharyngeal CXCL10 concentration and viral load consistent with our prior work, regardless of age, sex, race, ethnicity or virus identity (Fig. S1a–f).^16^^,^^19^^,^^20^ The receiver operating characteristic (ROC) curve describing biomarker performance to predict virus detection, show a similar area under the curve (AUC) in children (0.90, 95% C.I. 0.87–0.93; cohort 1), and adults (0.83, 95% C.I. 0.78–0.88, cohort 2), with overall AUC for all samples of 0.87 (95% C.I. 0.85–0.90) (Fig. 1b). There was also no significant difference in AUC when samples were compared by race, sex, ethnicity, or virus (Fig. S2). These results highlight similar biomarker performance in different patient groups and provide a rationale for using the combined dataset (n = 1088) for mathematical modelling to further assess test performance.

There was no significant difference between NP CXCL10 level in virus-negative samples and virus-positive samples with very low viral load based on PCR cycle threshold value (Ct value > 35, Fig. S3). Since the focus of this study was evaluating CXCL10 as a screening test for infection control, we used a Ct cutoff of <35 to define virus-positive samples for downstream analyses, consistent with prior studies showing that SARS-CoV-2 samples with Ct values higher than 35 do not contain infectious virus.^27^

**Fig. 2:**
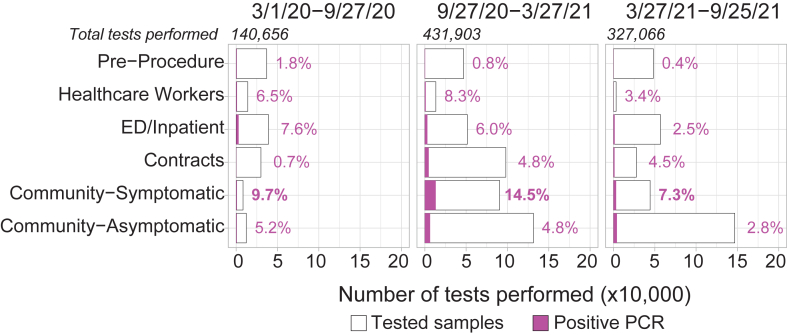
**Yale New Haven Hospital SARS-CoV-2 tests and positivity rates by test indication**. Number of SARS-CoV-2 PCR or NAAT tests done at YNHH for 6-month time intervals and their associated positivity rate in either asymptomatic, symptomatic, ED/inpatients, tests done for other institutions largely for asymptomatic screening (contracts), healthcare workers or pre-procedure patients. The study period included tests performed from week 10 of 2020 to week 38 of 2021. Purple bars and percentages indicate percentage of positive tests within each category.

Rule-out screening tests require a high negative predictive value (NPV), which is a function of both the test and viral prevalence in the population being screened. To estimate variation in viral prevalences among different populations undergoing respiratory virus screening, we examined SARS-CoV-2 testing at YNHH from March 2020 to September 2021, when PCR and other nucleic acid amplification tests (NAAT) were the only screening tests available (Fig. 2).^25^ YNHH performed 899,625 NAAT tests with an overall positivity rate of 5.04%. We further examined three timeframes: March–September 2020 (n = 140,656; 4.42% positive tests), October 2020–March 2021 (n = 431,903; 6.67% positive tests), and April 2021–September 2021 (n = 327,066; 3.14% positive tests). SARS-CoV-2 prevalence during these periods ranged from 2.8–5.2% for asymptomatic individuals, and from 7.3–14.5% for symptomatic, based on ask-at-order-entry questions which identified test indication. Since these data reflect testing for one virus only, we also considered pre-pandemic data based on multiplex testing for all common respiratory viruses. Prior work using symptom-agnostic testing showed virus positivity rates of ∼6% for adults in families with young children, whereas among adults with clinical indications for respiratory virus testing at YNHH, positivity rate was ∼30%, similar to that seen in our sample set (Fig. 1a).^28^^,^^29^

**Fig. 3:**
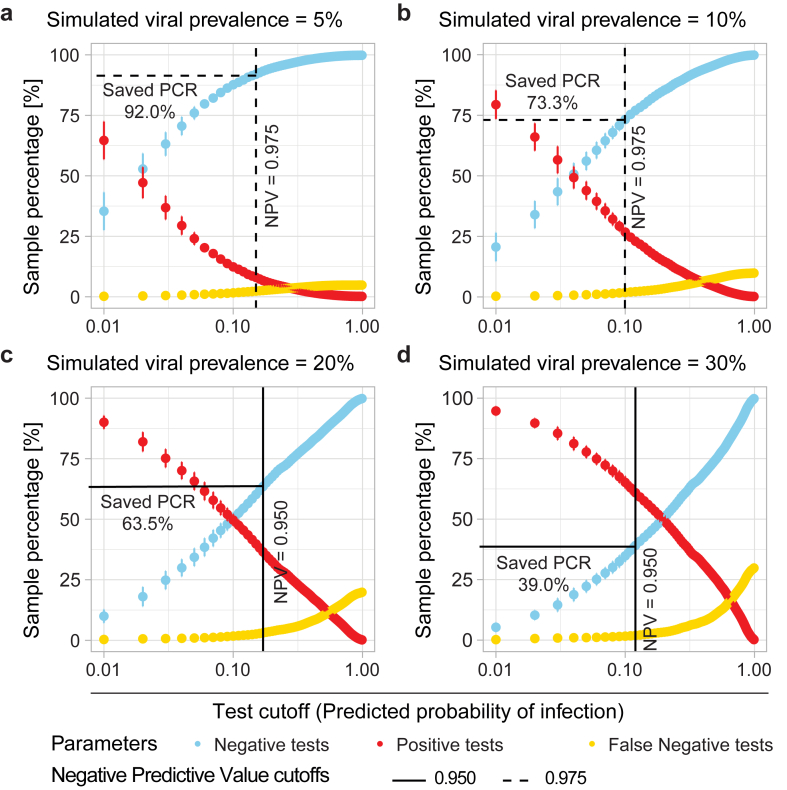
**Predicted relationship between the number of tests saved and the proportion of false negative tests in populations with different viral prevalences**. (**a–d)** Test performance at simulated viral prevalence of 5% (a), 10% (b), 20% (c) or 30% (d). Each of the subsampled data sets were fit to a logistical regression model and the entire range of test cutoffs (by 0.01 increments) was tested to estimate the percentage of positive (red), negative (blue) and false negative (yellow) tests at each cutoff. The process was repeated over a thousand bootstraps and graphs show mean value ± SD (coloured dots and vertical lines). Black vertical lines highlight NPV = 0.975 (dashed line) or 0.95 (solid line), while horizontal lines show the corresponding proportion of patients ruled out (displayed as saved PCR tests).

We next used random sub-sampling of the combined dataset (n = 1088) to model the effect of viral prevalence on test performance. Samples were first categorised as virus positive or virus negative based on PCR results. Then random subsampling was performed on each subset to create simulated populations at different viral prevalences (5%, 10%, 20%, or 30%), based on our exploration of virus positivity rates in different populations undergoing respiratory virus testing (Fig. 2). We then used a logistic regression model in which virus positivity by PCR was the response variable, and log_10_-transformed CXCL10 level was the predictor variable, to calculate the percentage of negative tests (blue line), positive tests (red line), and false negative tests (yellow line) across the full range of test cutoffs (Fig. 3a–d). As expected, both the total negative tests and false negative tests increased as the test cutoff increased (x-axis, Fig. 3a–d). Since the goal of a rule-out test is to predict virus negative status, we evaluated how screening would affect resource utilization using stringent cutoff values to minimise false negatives, equivalent to NPV of 0.975 or 0.95. At an NPV of 0.975, CXCL10 screening was predicted to result in 92% of samples testing negative when viral prevalence is 5% and 73.3% of samples testing negative when viral prevalence is 10%. When simulated viral prevalence was higher (20 or 30%), we used a cutoff of NPV 0.95 since few virus-negative subjects could be identified at NPV of 0.975. 63.5% or 39% of samples were predicted to test negative at viral prevalences of 20% or 30%, respectively (Fig. 3a–d and Table 1). To evaluate CXCL10 diagnostic performance in more detail, we calculated sensitivity and specificity at two test cutoffs, associated with best balanced accuracy or high sensitivity needed for a screening test (Fig. S4). This showed a sensitivity and specificity of 0.82 and 0.80, respectively, at the balanced cutoff and a sensitivity and specificity of 0.95 and 0.47 at the “high sensitivity” cutoff corresponding to NPV = 0.95 for the 1088 sample dataset (viral prevalence 32.6%). These values illustrate that at typical test cutoffs used for screening designed to maximise NPV, while a negative test rules out viral infection, a positive screening test does not rule in viral infection. However, subsampling to simulate typical prevalences for populations undergoing screening demonstrates the potential for biomarker-based pre-screening to greatly reduce the number of individuals requiring additional diagnostic testing, especially when viral prevalence is low. Fig. 3a–d also illustrate the inverse relationship between the number of individuals testing negative on a screening test, representing resource and time savings, and the potential for false negative tests, with the trade-off becoming more significant as the viral prevalence in the screened population increases.

**Table 1:** Estimation of resources saved by CXCL10 pre-screening.

Viral prevalence	Negative predictive value	PCR tests saved (%)	False negative (%)	Cost saving (%)		Cost saving for 10,000 patients tested (USD)	
				1:5^a^	1:15^b^	1:5^a^	1:15^b^
5%	0.975	92.0	2.3	72.0	85.3	331,500	1,174,000
10%	0.975	73.3	1.8	53.3	66.7	246,000	918,000
20%	0.950	63.5	3.1	43.5	56.8	192,000	756,000
30%	0.950	39.0	1.9	19.0	32.3	88,500	445,500

Resource utilization and cost analysis estimating the PCR tests saved (% CXCL10 biomarker-negative samples) and reduction in costs at different viral prevalences, when using CXCL10 screening followed by PCR testing on only the biomarker-positive samples.

USD = U.S. dollars.

a $9 for biomarker, $45 for PCR test.

b $9 for biomarker, $135 for PCR test.

**Fig. 4:**
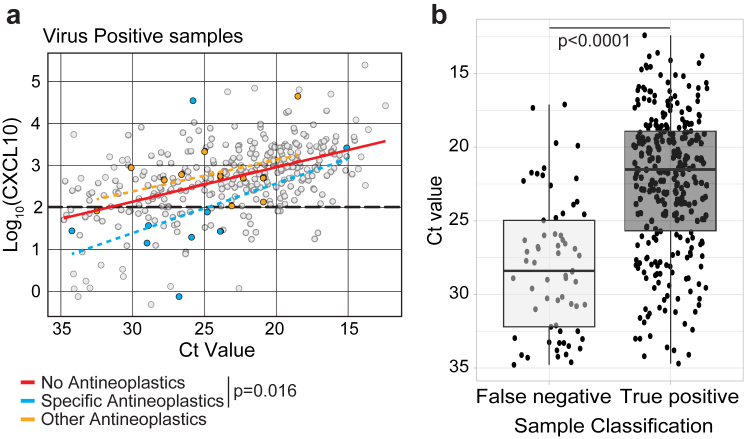
**Clinical and virological features distinguishing false negative from true positive samples**. **(a)** Linear regression depicting the correlation between Ct value obtained by RT-qPCR and log_10_-transformed CXCL10 concentrations for the following groups: patients treated with specific antineoplastics identified by outlier analysis (anthracyclines, taxanes, JAK inhibitors or vinca alkaloids) (blue), patients treated with other antineoplastics (gold), or patients not treated with antineoplastics (red line; grey dots). Regressions were compared for no antineoplastics vs. specific antineoplastics groups, which showed a significant difference in y-intercept (p = 0.016). (**b)** Comparison of viral load based on PCR Ct value in false negative (n = 65) and true positive (N = 290) samples based on a low stringency cutoff of 102 pg/mL corresponding to an NPV of 0.90 in the combined dataset. Graph shows median and interquartile range for each group. Statistical analysis was performed using student t-test.

Next, to understand the epidemiological significance of false negatives, and to identify possible exclusion criteria for CXCL10 screening, we sought to identify clinical characteristics associated with false negative tests. First, we performed an exploratory outlier analysis by comparing clinical metadata associated with samples testing positive for viruses by CXCL10 concentration and PCR (“true positives”) to samples testing negative by CXCL10 but positive by PCR (“false negatives”) (Fig. S5). To be as inclusive as possible of features which might impact test performance, we defined true negatives and false positives using a CXCL10 cutoff corresponding to NPV = 0.90. Comparisons of medication use revealed a possible association of false negative samples with five classes of anticancer chemotherapeutics: anthracyclines, taxanes, janus kinase (JAK) inhibitors, vinca alkaloids, and pyrimidine analogues (p-value≤0.001, (Chi-squared and student's t-test) (Table S2). To further evaluate whether these drugs were associated with a lower-than-expected concentration of CXCL10 based on viral load, we compared the relationship between Ct values obtained by RT-qPCR and NP CXCL10 concentration in samples from patients on these drugs to samples from patients on no antineoplastic drugs (Fig. 4a). Both regression lines had similar slopes, but the y-intercepts were significantly lower for patients on specific antineoplastics identified by outlier analysis (0.80 vs. 1.73 for patients on no anti-neoplastic drugs, p = 0.016, (ANCOVA)). This indicates lower than expected nasal CXCL10 concentration across the range of viral loads for patients on these drugs, supporting the result from the exploratory outlier analysis. These five classes of chemotherapeutics represented 45% (9/20) of the total patients on neoplastic drugs in the dataset. For patients on other chemotherapeutic drugs, there was no difference from the larger dataset in the relationship between Ct values and nasopharyngeal CXCL10 (Fig. 4a, gold symbols). Next, we explored other clinical features such as patient test results and diagnoses (Table S3). This analysis revealed only one feature linked to false negatives compared to true positives: low viral load based on the RT-qPCR Ct value. Supporting this result, median viral load was ∼80-fold higher for true positives compared to false negatives (median Ct value of 22.1 (S.D. 4.9) and 28.4 (S.D. 4.6), respectively, p = 1.6.10^−14^ (student's t-test); Fig. 4b).

Finally, in addition to enabling efficient triage, CXCL10 screening has the potential to impart cost savings by reducing the need for viral PCR testing in virus-negative subjects. Cost savings are expected to be highest when viral prevalence is low, since savings are counterbalanced by the need to do both biomarker testing and PCR on subjects who test positive on the screening test. Based on pricing of existing commercial PCR and immunoassay tests in the GSA Advantage database in the U.S., we estimate that the costs of a commercial PCR test range from 5 to 15-fold higher than the cost of a commercial point-of-care immunoassay, depending on whether PCR testing is done for a single respiratory virus, a limited panel of four common viruses, or a comprehensive panel of 10+ viruses.^30^ Table 1 summarises the estimated cost savings for a strategy in which all subjects are pre-screened using the biomarker immunoassay, and only those with CXCL10 above the cutoff undergo viral PCR testing. We estimated the cost savings per 10,000 tests, the approximate number of tests performed weekly in our healthcare system between March 2020 and September 2021 (Fig. 2). At a viral prevalence of 5% and NPV of 0.975, CXCL10 screening would be expected to reduce overall costs by 72%–85.3%. Taken together, these results indicate that in addition to rapid diagnostic discrimination for triage, biomarker-based screening could enable significant resource and cost savings in situations that require frequent or widespread screening for viral respiratory infection.

## Discussion

During a respiratory virus epidemic or in other settings which require strict infection control, rapid, cost-effective methods for ruling out viral respiratory infection could have a large positive impact by (1) reducing infections, morbidity, and mortality from viral spread, (2) reducing the need for isolation of uninfected individuals, and (3) reducing costs and directing limited healthcare resources for those at greatest risk of infection. Given the low accuracy of symptom-based screening, and the benefit of virus-agnostic testing inclusive of novel viruses, we propose a strategy of host biomarker-based screening, followed by additional testing of samples that are biomarker-positive.

Our prior study identified nasopharyngeal CXCL10 as a biomarker of viral respiratory infection using samples from ∼200 symptomatic subjects from 2017 to 18, who were mostly adults over 50 years of age.^16^ Here we used a larger and more heterogeneous sample set with a roughly equal number of samples from adults and children, with a diverse self-reported racial and ethnic composition representative of the patient population of the Yale–New Haven healthcare system which serves southern Connecticut and eastern New York state in the United States. We found similar diagnostic accuracy across these categories (AUC in prior study 0.87, 95% C.I. (0.81–0.93); AUC in this study 0.87, 95% C.I. (0.85–0.90), supporting the usefulness of this test across diverse patient groups, although it will be important to evaluate this further in future studies with global populations with different racial/ethnic compositions and different environmental or microbial exposures. We also found no significant difference in the relationship between nasopharyngeal CXCL10 concentration and viral load for different viruses, although we cannot rule out subtle differences given that sample size was limited for individual viruses. This study also included samples from asymptomatic individuals. While there were not enough virus-positive asymptomatic subjects to examine test performance in asymptomatic subjects only, we previously showed that nasal CXCL10 concentration directly correlates with viral load in both asymptomatic and symptomatic subjects, supporting its utility as a screening test.^20^ For SARS-CoV-2, studies of both natural and experimental infections document that infection and high nasal viral loads can occur in asymptomatic individuals, including prior work from our group describing individuals who had no symptoms or mild symptoms but high nasal SARS-CoV-2 viral loads and elevated nasal CXCL10.^6^, ^7^, ^8^^,^^31^ We hypothesise that the robust performance of CXCL10 as a biomarker and its direct correlation with viral load is due to its strong induction by innate immune sensing of viral RNA and amplification by interferon during the early host antiviral response at the site of infection.^16^^,^^32^ However, given this biology, CXCL10 may have limited sensitivity for early stage, low viral load infections. While low nasal viral loads are much less likely to result in transmission than high nasal viral loads, early low viral load infection can develop into a high viral load infection. A possible solution to missing early-stage infections is regular re-testing, as recommended for the at-home COVID-19 antigen tests which have the same limitation. Along these lines, while this study utilised nasopharyngeal swabs, adapting this biomarker to less invasive sample types, such as nasosorption or anterior nares swabs, will further facilitate use as a frequent screening test and is a future direction of this work.

To implement CXCL10 as a screening test, it is also important to consider clinical features which may affect test performance. Systematic comparison of medication use and follow-up analyses identified a subset of chemotherapeutic drugs associated with reduced nasopharyngeal CXCL10 response to viral respiratory infection, suggesting that CXCL10 may be a less sensitive indicator of viral infection for patients on these medications. Another feature associated with false negative results was low viral load. In addition to very early infections discussed above, resolving infections with some respiratory viruses are associated with persistent virus detection by qPCR at high Ct values (low viral loads). Research on SARS-CoV-2 indicates that PCR positivity 8 days after symptom onset is associated with negligible infectivity. While further work will be needed to determine whether persistent virus detection by PCR is clinically significant for other viruses, evidence to date suggests that persistent low-positive PCR tests during resolving infections may not be significant for infection control.^27^ In addition to the specific findings, the outlier analysis done here demonstrates the benefits leveraging large scale EHR data to reveal subtle unappreciated associations difficult to discern by conventional approaches, which can then be prospectively evaluated in subsequent studies.

For a screening test, both the NPV, corresponding to accurate triage, and the number of samples testing negative, corresponding to resource savings, are highest when the viral prevalence is low. For example, in our health care system, from March 2020 to September 2021, 422,642 SARS-CoV-2 PCR screening tests were performed on asymptomatic subjects in the community (including pre-procedure patients) but most of these tests were negative (97%; 410,318/422,642). Our mathematical modelling and cost analysis results indicate that if CXCL10 pre-screening had been implemented, >92% of these PCR tests could have been avoided. This example, based on real-world data, illustrates how a biomarker-based screening test could have helped to mitigate supply chain pressure, reduce costs, and conserve valuable resources during the first year of the COVID-19 pandemic.

Importantly, at the cutoffs described in this paper, a negative CXCL10 test would be useful for ruling out viral infection but a positive CXCL10 test would not rule in viral infection. Due to the tradeoff between sensitivity and specificity, setting the test cutoff to have high sensitivity (reduce false negatives) is expected to lower specificity (increase false positives), therefore CXCL10-high samples would be reflexed to PCR testing in our proposed diagnostic algorithm. Other types of infection or inflammation could potentially lead to an elevated nasal CXCL10 concentration, such as infection with other microbes or allergic inflammation. Our prior work showed that viral infection is a much stronger driver of nasal mucosal CXCL10 than common upper respiratory bacteria, ruling out one important potential confounder.^20^ We also investigated causes of nasal CXCL10 elevation in patients testing PCR negative for a panel of seasonal respiratory viruses in prior work, which identified unexpected viruses (e.g. SARS-CoV-2 at the start of the COVID-19 pandemic), primary EBV or CMV infection, and sepsis with cytokine storm as causes of high nasal CXCL10.^19^ Future systematic analysis of non-viral causes of CXCL10 elevation will enable further refinement of this screening strategy.

This study has several limitations. First, our estimates for viral prevalence during the COVID-19 pandemic are based on SARS-CoV-2 testing alone. While circulation of some common seasonal viruses (e.g. influenza, RSV), fell during the early pandemic due to public health interventions there is evidence that other seasonal viruses continued to circulate, particularly in young children.^33^ This study's results imply that CXCL10 screening would offer the greatest advantage for populations with low rates of viral respiratory infections, such as asymptomatic adults (expected viral prevalence <10%) and would be least useful for screening populations with high virus positivity rates (e.g. young children).

Another caveat is limitations of the dataset. Although >1000 samples were evaluated, our dataset originates from patients seeking medical attention for respiratory symptoms or other reasons, and test performance could be different in a largely healthy population. Similarly, rare pathologies or treatments might not be represented and therefore might not be captured by the outlier analysis. Since we did not have detailed clinical information for many samples, we could not assess whether clinical presentation influences test performance. Also, due to the timing of sample collection, this study did not include SARS-CoV-2 positive samples from adults, although our prior work on SARS-CoV-2 positive adults shows that NP CXCL10 is elevated in the nasopharynx in direct proportion to viral load, similar to other viruses.^19^^,^^31^ This was also a retrospective study using previously frozen samples for biomarker measurements. Studying specific cohorts and testing fresh samples prospectively has the potential to further improve test performance, as does refining the testing strategy for specific clinical scenarios.

In conclusion, this study demonstrates the usefulness of host biomarker-based screening as a new tool in the armamentarium for management of viral outbreaks during epidemics, or to prevent outbreaks of seasonal respiratory viruses in confined settings such as nursing homes, health care facilities, dormitories, and military barracks. Experience with use in real world settings will further facilitate this strategy to reduce respiratory virus transmission and advance readiness for a future pandemic.

## Contributors

Data curation J.A., S.D., N.C, T.W., A.G., P.Y., D.P., M.L., W.S., E.F.; Formal analysis J.A., S.D., W.S., E.F., Investigation J.A., S.D., N.C., T.W., A.G., Software J.A., S.D., W. S., Visualization J.A., S.D., Resources M.L., W.S., Conceptualization E.F., Funding Acquisition E.F., Methodology, all authors, Writing—Original Draft J.A. and E.F.; Writing—reviewing and editing, all authors. J.A. and E.F. accessed and verified all test performance data and S.D. and W.S. accessed and verified all clinical and outlier analysis data reported in the study. E.F. was responsible for the decision to submit. All authors reviewed and approved the final version of the manuscript.

## Data sharing statement

The original code and data for test performance analyses are publicly available in Mendeley Data at https://doi.org/10.17632/kxp7wxpyvb.1 (Amat and Foxman, 2025). Python scripts used for the clinical data extraction and outlier analysis are available with appropriate data use and security agreements upon request to wade.schulz@yale.edu.

## Declaration of interests

Dr. Foxman and Dr. Landry are inventors on U.S. Patent 11,965,218. Dr. Foxman is an inventor on U.S. Patent 12,110,564 and pending patent application PCT/US22/82314. Dr. Schulz is a consultant for Hugo Health, consultant for Detect Inc, a point-of-care diagnostics company; co-founder of Refactor Health, and has received research funds from Merck and Regeneron. Dr. Peaper has received research funding from Hologic, Inc and Honoria from Becton–Dickinson. The other authors declare no competing interests.
