# Supplementary material for: Nasal biomarker testing to rule out viral respiratory infection and triage samples: a test performance study
Source: eBioMedicine. 2025 Jun 20;117:105820. doi: 10.1016/j.ebiom.2025.105820 (PMC12216728; doi:10.1016/j.ebiom.2025.105820)
Supplement: Supplementary Figures and Tables [file mmc1.pdf]

Supplementary Documents:

Nasal biomarker testing to rule out viral respiratory infection and triage samples: a test performance study

Supplementary Figures

Fig S1. Relationship between nasal CXCL10 concentration and qPCR Ct Value by patient demographic group or virus……………………………………………………………………………………………………….2

Fig S2. Receiver operating characteristic (ROC) curves for predicting virological status based on log_{10}-transformed CXCL10 protein concentrations by patient demographic and virus group………………….......................................4

Fig S3. Relationship between the nasal CXCL10 concentration and qPCR Ct value subgroups………………………5

Fig S4. Receiver operating characteristic (ROC) curves and associated specificity and sensitivity at set cutoffs…….6

Fig S5. Strategy for outlier analysis…………………………………………………………………………………7

Supplementary Tables

Table S1. Patient demographics……………………………………………………………………………….….8

Table S2 (Excel file). Medication comparison between true positive and false negative groups. Comparison of frequency of prescription drugs between true positive and false negative populations for drug classes identified by ATC code.

Table S3 (Excel file). Clinical condition comparison between true positive and false negative groups. Comparison of frequency of clinical conditions between true positive and false negative populations for each unique diagnosis category identified by the first three digits of the ICD-10 code.

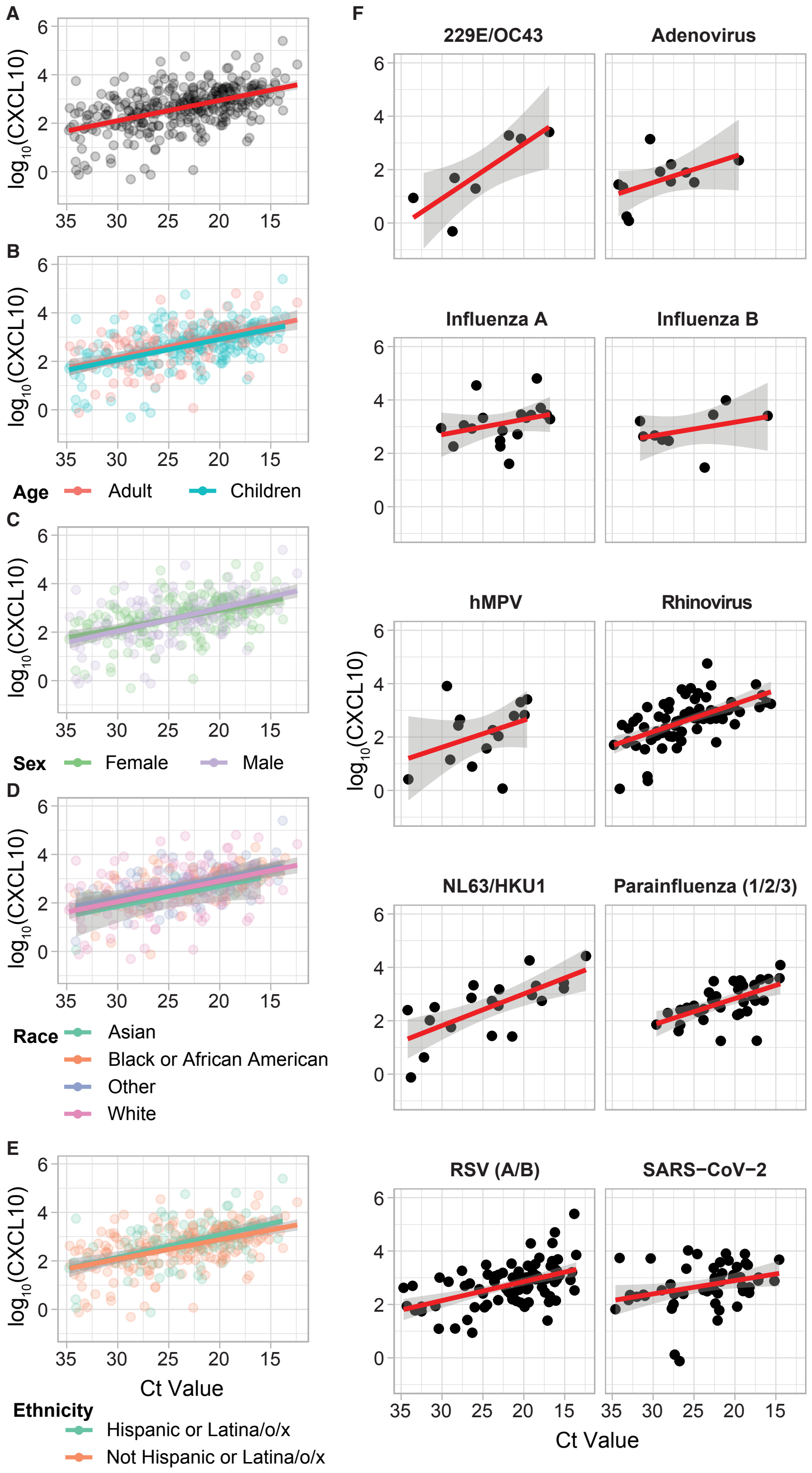

Figure S1. Relationship between nasal CXCL10 concentration and viral load by patient demographic group or virus.

(A) Correlation of Ct values obtained by RT-qPCR and the log_{10}-transformed CXCL10 concentrations for all virus positive samples. Linear regression is shown in red and 95% C.I. is depicted in grey.

(B–F) Correlation of viral load (35-Ct) values and the log_{10}-transformed CXCL10 concentrations of samples by age (B), sex (C), race (D) or ethnicity (E) or virus (F). Individual linear regressions are shown in red and blue (age classes), or purple and green (sex), or green, orange, purple and pink (race), or green and orange (ethnicity). 95%CI are depicted in grey. There was no significant difference in slope of regression line among sample subsets (ANCOVA test). Abbreviations: 229E/OC4, Seasonal coronaviruses 229E and OC43; hMPV, human metapneumovirus; NL63/HKU1, Seasonal coronaviruses NL63 and HKU1; RSV, respiratory syncytial virus

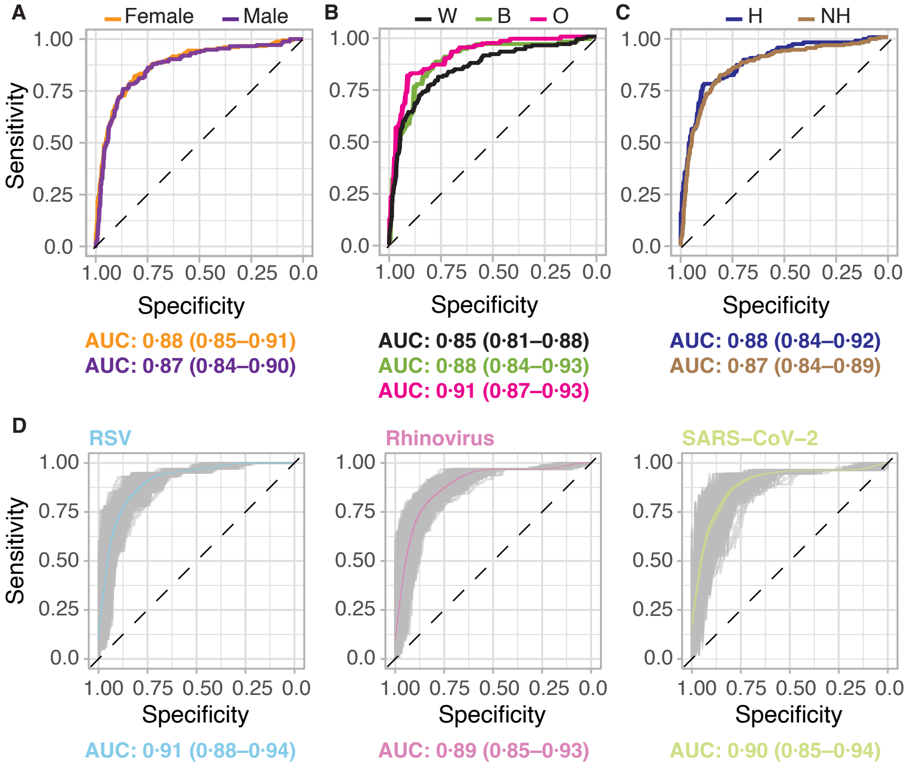

Figure S2. Receiver operating characteristic (ROC) curves for predicting virological status based on log_{10}-transformed CXCL10 protein concentrations in patient subgroups.

Subgroups with at least 200 subjects were included and are stratified by **(A)** biological sex (female (orange) and male (purple)), **(B)** self-reported race (White (W; black), Black (B; green), or Other (O; magenta)), and **(C)** self-reported ethnicity (Hispanic or Latina/o/x (H; navy blue) or Not Hispanic or Latina/o/x (NH; brown)). **(D)** ROC curves for individual viruses with at least 50 positives in the dataset including RSV (n=100, cyan), rhinovirus (n=95, pink), and SARS-CoV-2 (n=55, light green). ROC curves were generated by using random subsampling of virus PCR negative samples to create a balanced dataset with an equal number of positives and negatives for each virus. Individual ROCs (grey) of thousand iterations of the are shown, while mean and 95% C.I. are depicted as coloured lines. Area under the curve (AUC) with 95% C.I. are shown beneath each plot.

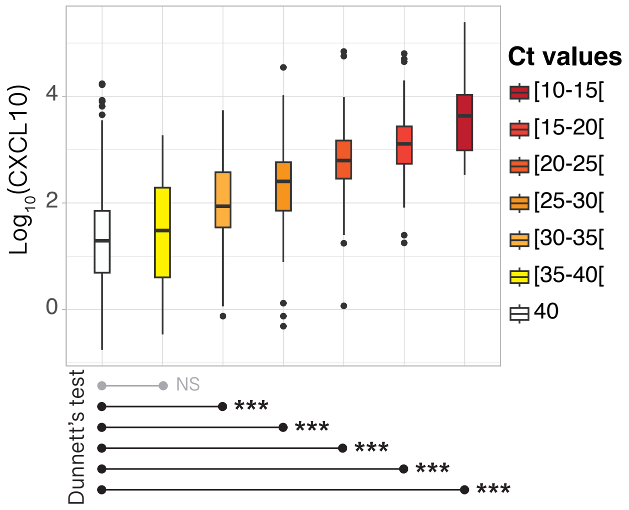

Figure S3. Relationship between the nasal CXCL10 concentration and viral load by Ct value. Boxplots depicting the distribution (median and interquartile range (IQR)) of the log_{10}-transformed CXCL10 concentration in different groups based on viral load (PCR cycle threshold (Ct) values). Each group was compared to Ct<40 using Dunnett's test.

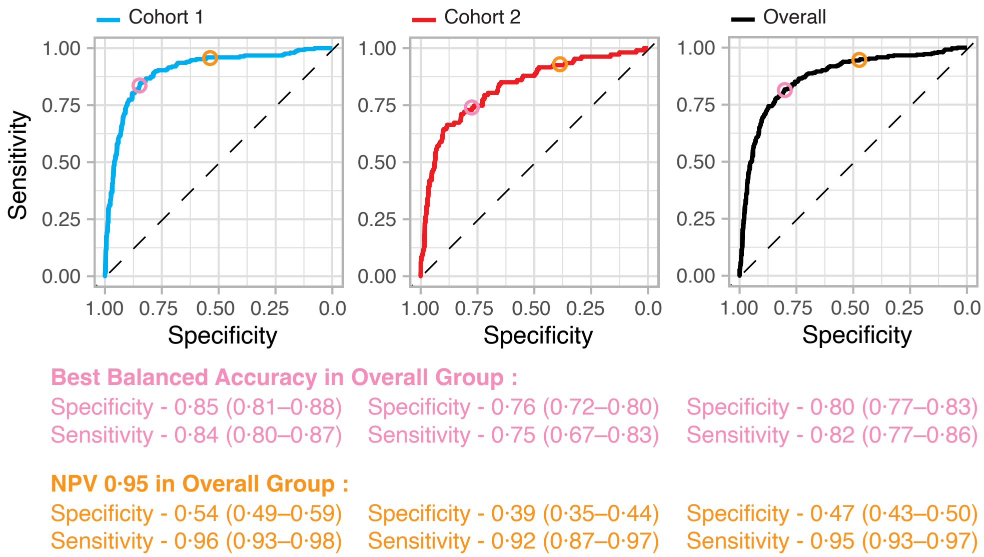

Fig S4. Receiver operating characteristic (ROC) curves and associated specificity and sensitivity at two cutoffs. Receiver Operating Characteristic (ROC) curves depicting the prediction of virological status based on the $\log_{10}$-transformed CXCL10 protein concentration in cohorts 1 and 2 shown in blue and red respectively or combined (black). Sensitivity and specificity with 95% C.I. are shown for test cutoffs with best-balanced accuracy (magenta) or high sensitivity (orange) equivalent to an overall NPV = 0·95 for the 1088 sample dataset.

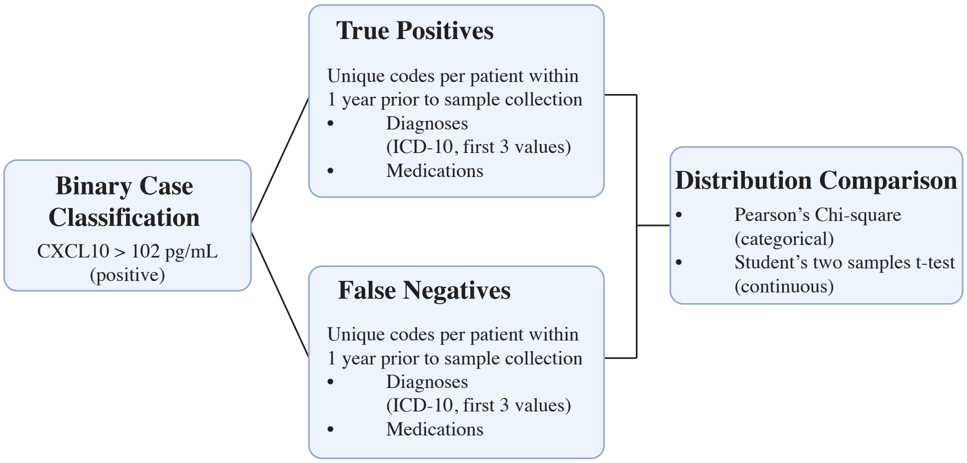

Fig S5. Strategy for outlier analysis. Flowchart indicating the workflow used to identify variables were found with different frequencies in true positive and false negative populations**.** After the distribution comparison, a domain expert reviewed statistically significant and clinically relevant variables for downstream exploratory analysis.

	Number of patients	
	**Cohort 1 (Children)**	**Cohort 2 (Adults)**
**Sex**		
Female	287 (48·5%)	251 (50·6%)
Male	305 (51·5%)	245 (49·4%)
**Race**		
Asian	21 (3·6%)	7 (1·4%)
Black or African American	128 (21·6%)	98 (19·8%)
White	277 (46·8%)	334 (67·3%)
Other	166 (28·0%)	57 (11·5%)
**Ethnicity**		
Hispanic or Latino/a/x	207 (35·0%)	65 (13·1%)
Non-Hispanic or Latino/a/x	375 (63·3%)	426 (85·9%)
Other	10 (1·7%)	5 (1·0%)
**Total**	592 (100%)	496 (100%)

Table S1. Patient demographics. Number and percentage of study subjects by sex, race, and ethnicity. Cohort 1 denotes children and cohort 2 denotes adults.
